# Linking genomic and physiological characteristics of psychrophilic *Arthrobacter* to metagenomic data to explain global environmental distribution

**DOI:** 10.1186/s40168-021-01084-z

**Published:** 2021-06-12

**Authors:** Liang Shen, Yongqin Liu, Michelle A. Allen, Baiqing Xu, Ninglian Wang, Timothy J. Williams, Feng Wang, Yuguang Zhou, Qing Liu, Ricardo Cavicchioli

**Affiliations:** 1 State Key Laboratory of Tibetan Plateau Earth System and Resources Environment, Institute of Tibetan Plateau Research, Chinese Academy of Sciences, Beijing, 100101 China; 2 College of Life Sciences, Anhui Normal University, Wuhu, 241000 China; 3 Center for the Pan-third Pole Environment, Lanzhou University, Lanzhou, 730000 China; 4 School of Biotechnology and Biomolecular Sciences, University of New South Wales, Sydney, NSW 2052 Australia; 5 College of Urban and Environmental Science, Northwest University, Xian, 710069 China; 6 China General Microbiological Culture Collection Center (CGMCC), Institute of Microbiology, Chinese Academy of Sciences, Beijing, 100101 China

**Keywords:** Genomics, Metagenomics, Psychrophiles, Polar environment, Alpine environment, Microbial adaptation

## Abstract

**Background:**

Microorganisms drive critical global biogeochemical cycles and dominate the biomass in Earth’s expansive cold biosphere. Determining the genomic traits that enable psychrophiles to grow in cold environments informs about their physiology and adaptive responses. However, defining important genomic traits of psychrophiles has proven difficult, with the ability to extrapolate genomic knowledge to environmental relevance proving even more difficult.

**Results:**

Here we examined the bacterial genus *Arthrobacter* and, assisted by genome sequences of new Tibetan Plateau isolates, defined a new clade, Group C, that represents isolates from polar and alpine environments. Group C had a superior ability to grow at −1°C and possessed genome G+C content, amino acid composition, predicted protein stability, and functional capacities (e.g., sulfur metabolism and mycothiol biosynthesis) that distinguished it from non-polar or alpine Group A *Arthrobacter*. Interrogation of nearly 1000 metagenomes identified an over-representation of Group C in Canadian permafrost communities from a simulated spring-thaw experiment, indicative of niche adaptation, and an under-representation of Group A in all polar and alpine samples, indicative of a general response to environmental temperature.

**Conclusion:**

The findings illustrate a capacity to define genomic markers of specific taxa that potentially have value for environmental monitoring of cold environments, including environmental change arising from anthropogenic impact. More broadly, the study illustrates the challenges involved in extrapolating from genomic and physiological data to an environmental setting.

Video Abstract

**Supplementary Information:**

The online version contains supplementary material available at 10.1186/s40168-021-01084-z.

## Background

Many biotic and abiotic factors influence the ability of microorganisms to become indigenous members of environmental communities. Certain environmental factors can limit or prevent the growth of microorganisms, while enhancing, or being essential for others, resulting in ecological niches that support specific microbiome structures [1]. This phenomenon is well illustrated by a Winogradsky column where light and oxygen can be seen to exert major influences on the diversity and the dynamic of microorganisms throughout its length [2]. In more recent times, particularly through technological advancement (e.g., metagenomics), the understanding of microbial ecology and the contributions that microorganisms make to the natural world has grown considerably. Appreciation for microorganisms has accrued from discoveries of new biomes capable of supporting microbial colonization, such as the deep subsurface [3]; new examples of life hidden within microbial “dark matter” (e.g., Asgard archaea [4]); and the dynamic nature of microbial responses, particularly those that provide surprises, such as the major societal upheaval caused by the SARS-CoV-2 coronavirus. There is a growing realization that microorganisms constitute the life support system of the biosphere and must be properly accounted for when devising strategies to mitigate the impacts of human activity on the natural world [5]. In essence, we are living in a period in history when the need for society to learn about microbial responses to natural and anthropogenic influences is of unprecedented relevance [5–7].

Metagenomic methods have provided a level of insight into microbial communities [8, 9] that could possibly be equated to the advances made by Carl Woese and colleagues when using rRNA sequencing to discover Archaea as the third domain of life. Applied to the cold biosphere, Earth’s single largest biome, metagenomic analyses have catalogued diverse ways in which microbial life has evolved. As an example, Antarctic, marine-derived lake communities have been shown to have evolved independently over their relatively short history of 3000–5000 years, adapting not just to low temperature but also to a variety of important environmental factors specific to each lake system (reviewed in Ref. [10]). Metagenomic analyses have also begun to be used to uncover the ways in which communities in polar environments respond to changing environmental conditions; for example, the effects of the seasonal polar sunlight cycle on Antarctic marine and marine-derived lake communities [11, 12] and the roles that Arctic bacteria play in melting permafrost acting as a CO_2_ source and atmospheric CH_4_ sink [13, 14].

*Arthrobacter* (Actinobacteria; Micrococcales; Micrococcaceae) are a globally distributed genus of bacteria commonly found in soil, but also in a broad range of environments including water, human skin, and sewage [15–18]. *Arthrobacter* are reported to play important roles in global biogeochemical cycles and decontamination of polluted environments [17, 19]. Their responses to temperature, desiccation, ionizing radiation, oxygen radicals, and a range of chemicals have been described [20–22]. Their growth in the laboratory is characterized by nutritional versatility that translates to an ability to grow aerobically in media utilizing a wide range of carbon and nitrogen sources [16]. Some isolates closely related to the type species *A. globiformis* were obtained from a Lapland glacier region [23], and the identification of psychrophilic species has led to the characterization of a number of *Arthrobacter* enzymes for their biotechnological potential (e.g., Ref. [24]). *Arthrobacter* have been isolated from a range of low-temperature environments, including permafrost and glaciers [25–27].

Due to the large scale of the Earth’s cold biosphere and its relevance to global biogeochemical cycles, and the biotechnological potential of psychrophiles and their products, numerous studies have been performed to attempt to define the critical traits of psychrophiles (discussed in Ref. [10, 24, 28–34]). In the current study, we sequenced the genomes of *Arthrobacter* isolated from lakes, glaciers, and a wetland from the Tibetan Plateau (Additional file 1: Fig. S1) and utilized the existence of more than 100 *Arthrobacter* genomes to assess traits that may explain the presence of the genus in naturally cold environments. After identifying a clade characteristic of polar and alpine environments and determining that representatives had a superior ability to grow at low temperature, we used available metagenome data to assess the environmental relevance of the findings. What we learned illustrated the complexities involved in attempting to extrapolate from genomic and physiological data to an environmental setting. It also revealed possible avenues for utilizing *Arthrobacter* as biomarkers of environmental warming.

## Results

### Phylogenomics

To increase the number of *Arthrobacter* genomes from *p*olar and *a*lpine (PA) environments, a total of 16 isolates from seven lakes, two glaciers, and one wetland on the Tibetan Plateau (Additional file 1: Fig. S1 and Table S1) were sequenced (see the “Methods” section). After genome quality assessment and dereplication, 13 high-quality genome sequences for new Tibetan Plateau isolates were incorporated into the study (Additional file 2: Dataset S1). The phylogenomic relationships of a total of 210 non-redundant high-quality Micrococcaceae genomes (Additional file 2: Dataset S1) were analyzed by constructing maximum likelihood and Bayesian trees (Additional file 1: Fig. S2). The two trees were congruent and most tree-nodes (194/208) were supported by high bootstrap values (>70%) (Additional file 1: Fig. S2). The *Arthrobacter* lineage formed a cluster with 106 representatives that was clearly separated from other Micrococcaceae genera (Additional file 1: Fig. S2). The *Arthrobacter* genomes represented PA isolates (total 31, including the 13 new genomes), with the remainder from a broad range of *n*on-*p*olar or *a*lpine (NPA) environments (Additional file 2: Dataset S1).

**Fig. 1:**
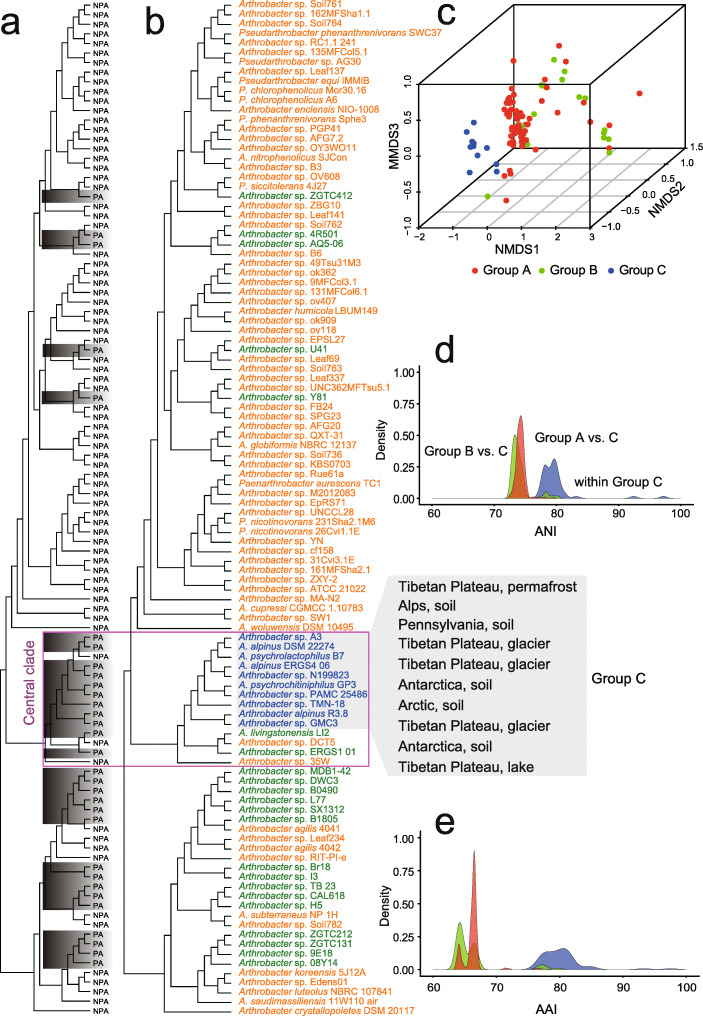
*Arthrobacter* phylogeny and genome compositional profiling. **a** Maximum likelihood *Arthrobacter* phylogenomic tree. The *Arthrobacter* portion of maximum likelihood Micrococcaceae phylogenomic tree (Additional file 1: Fig. S2a) is reproduced with each leaf marked as polar and alpine (PA, gray highlight) or non-polar and alpine (NPA). The tree has three major clades with the central clade highlighted (purple box). **b** As for **a** except *Arthrobacter* names denoted and font color used to depict Group A (orange font; NPA environments), Group B (olive green font; PA environments clustering with sequences from NPA environments), and Group C (blue font; PA environments that formed an operationally monophyletic lineage with an *F* measure of 0.95). The specific types of cold environments from where Group C *Arthrobacter* were isolated are shown to the right of the tree. **c** Three-dimensional nonmetric multidimensional scaling (NMDS) plot of genome-wide amino acid composition. **d** Distribution of pairwise average nucleotide identity (ANI). **e** Distribution of pairwise average amino acid identity (AAI)

The *Arthrobacter* lineage separated into three clades that branched from the root of the *Arthrobacter* tree (Fig. 1). The 31 PA *Arthrobacter* were distributed across the tree, although 11 PA *Arthrobacter* formed a cluster with three NPA *Arthrobacter* in the central clade (Fig. 1 a). Within the central clade, 10 *Arthrobacter* grouped together (Fig. 1 b, blue font) with an *F* measure of 0.95, defining them as an operationally monophyletic lineage [35]. This was supported by three-dimensional nonmetric multidimensional scaling analysis of amino acid composition (Fig. 1 c) and both average nucleotide identity (ANI) and average amino acid identity (AAI) distributions (Fig. 1 d, e). The grouping of 10 *Arthrobacter* (Fig. 1 b, blue font) consisted of nine PA *Arthrobacter*, plus *A. psychrolactophilus* B7 which was isolated from Pennsylvania soil following snow melt; the isolate was obtained as a source of cold-active enzymes and was capable of growth at 0 °C [36]. The monophyletic lineage of 10 *Arthrobacter* was defined as Group C (Fig. 1 b, blue font), with all other PA *Arthrobacter* as Group B (Fig. 1 b, olive green font), and all *Arthrobacter* from NPA environments as Group A (Fig. 1 b, orange font).

### Low-temperature growth capacity of Group C *Arthrobacter*

**Fig. 2:**
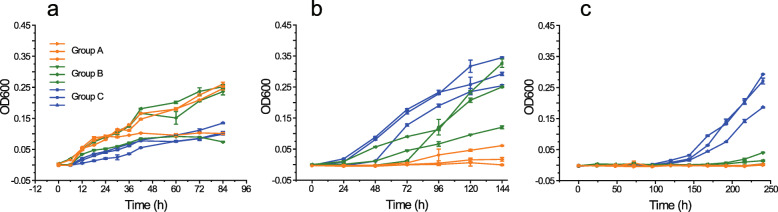
Growth temperature profiles of Group A, B, and C *Arthrobacter*. OD_600_ growth curves for representative *Arthrobacter* of Group A (orange symbols and line; *A. luteolus*, *A. globiformis*, and *A. subterraneus*), Group B (olive green symbols and line; *Arthrobacter* sp. 4R501, *Arthrobacter* sp. 9E14, and *Arthrobacter* sp. 08Y14), and Group C (blue symbols and line; *A. alpinus*, *Arthrobacter* sp. A3, and *Arthrobacter* sp. N199823) at **a** 25 °C, **b** 5 °C, and **c** −1 °C

To evaluate the growth temperature response of Group A, B, and C, three *Arthrobacter* from each group were grown at 25, 5, and −1 °C and growth monitored (OD_600_) over time (Fig. 2). The three Group C *Arthrobacter* exhibited a markedly enhanced rate of growth at −1 °C (Fig. 2 c) particularly compared to Group A *Arthrobacter* and had a reduced rate of growth at 25 °C compared to some of the Group A and B *Arthrobacter* (Fig. 2 a, b).

### Genomic characteristics

**Fig. 3:**
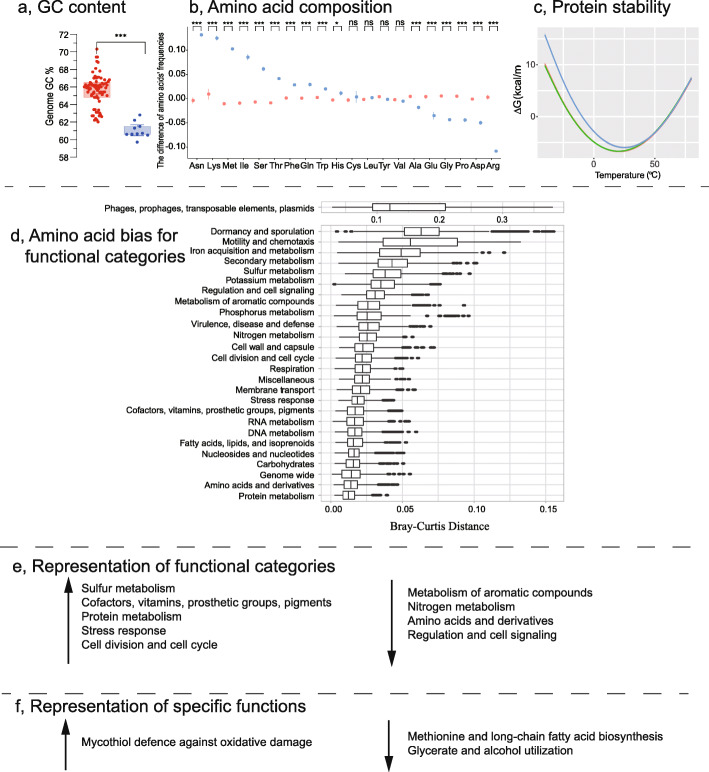
Overview of genomic characteristics of Group C *Arthrobacter*. **a** Box plot of G+C content. Group A (red); Group C (blue); boxes represent the interquartile range with horizontal lines showing maximum and minimum values, excluding outliers. Group C had significantly lower G+C content. **b** Scatter plot of amino acid composition. Group A (light red circles); Group C (blue circles); ***p < 0.005; *p = 0.05–0.01; ns, not significant. The composition of numerous amino acids varied significantly between Group C and Group A *Arthrobacter.* **c** Protein stability predictions calculated using SCooP. Group A (red line); Group C (blue line). The curve is for coenzyme A biosynthesis bifunctional protein, CoaC, and is representative of one of the 32 Group C proteins from a total of 86 which had reduced predicted stability (Additional file 3: Dataset S2 and Additional file 1: Fig. S6) **d** Box plot of amino acid bias for functional categories. Boxes represent the interquartile range of the Bray-Curtis distances; lines extending from boxes show the maximum and minimum Bray-Curtis distances; dots beyond the lines represent outliers. Biases in amino acid composition (**b**) were reflected in specific functional categories. **e** Representation of functional categories. Specific functional categories were over- or under-represented in Group C; arrows indicate relative increases (up arrow) or decreases (down arrow) in functional categories in Group C. **f** Representation of specific functions. Specific functional processes defined by genes or pathways were characteristic of Group C (up arrow) or had a restricted capacity in Group C (down arrow) compared to Group A (also see Fig. 4)

The size of the 106 *Arthrobacter* genomes ranged from 3.24 to 5.89 Mbp (Additional file 2: Dataset S1). Between Group A and C, no significant differences occurred in genome size, 16S rRNA and tRNA gene copy number, or coding density (Additional file 1: Fig. S3). However, a significant difference was observed in amino acid composition and G+C content (Fig. 3, Additional file 1: Fig. S3 and Additional file 2: Dataset S1). In Group C, the content of N, K, M, I, S, T, F, Q, W, and H was significantly higher, while A, E, G, P, D, and R was significantly lower (one-way ANOVA, *p* < 0.05, Additional file 1: Fig. S4). The correlation between amino acid composition and G+C content was significantly negative for the amino acids N, I, M, S, F, K, and Q (*R*^2^ ranged from 0.46 to 0.81; *p* < 0.01) and significantly positive for W, G, D, P, R, and A (*R*^2^ ranged from 0.39 to 0.77; *p* < 0.01) (Additional file 1: Fig. S5).

To evaluate the potential structural relevance of the amino acid compositional differences, temperature-dependent protein stability predictions were made using SCooP, which predicts stability assuming proteins are monomeric and follow a two-state folding transition [37]. From 180 proteins targeted for evaluation (mostly single-copy genes; see the “Methods” section), 86 produced robust stability curves (Fig. 3, Additional file 1: Fig. S6 and Additional file 3: Dataset S2). Stability was calculated at −1 °C to match the growth temperature at which Group C showed a marked difference in growth ability (Fig. 2). The 86 Group C proteins had significantly higher Δ*G* values (Group A, −4.27; Group B, −4.29; Group C, −3.60; *p* < 0.01), with 32 proteins being responsible for the reduced predicted stability (Additional file 1: Fig. S6 and Additional file 3: Dataset S2). These 32 proteins contained a particularly high representation of the amino acids that were most over-represented in Group C (i.e., N, K, and R; Additional file 1: Fig. S4). The 32 proteins represented 12 functional categories, primarily metabolism (28 proteins; 9 categories), with four involved in respiration, stress response, or cell division and cell cycle (Additional file 3: Dataset S2). The marked amino acid compositional differences, broad representation of functional categories, and high proportion of proteins with predicted decreases in stability (~ 1/3rd of those tested) demonstrate that Group C *Arthrobacter* possess broad genomic differences to Group A *Arthrobacter*. If the decreases in predicted protein stability translate to an increased capacity to perform catalysis at low temperature, this may contribute to the higher growth rates of Group C at −1°C (Fig. 2).

To further explore the influence of amino acid composition on functional potential, Bray-Curtis distances of genome-wide amino acid composition were evaluated for proteins representing 26 functional categories (Fig. 3). The greatest distance was for the category “phages, prophages, transposable elements, plasmids,” consistent with previous studies associating transposable elements with cold-adapted microorganisms [38–40]. The functional potential of Group C was also compared to Group A using enrichment analysis [41] performed on proteins representing the 26 functional categories. Group C was over-represented in sulfur metabolism; cofactors, vitamins, prosthetic groups, pigments; protein metabolism; stress response; cell division; and cell cycle (Fig. 3, Additional file 1: Fig. S7 and Additional file 4: Dataset S3), whereas Group A was over-represented in the metabolism of aromatic compounds; nitrogen metabolism; amino acids and derivatives; regulation; and cell signaling (Additional file 1: Fig. S7 and Additional file 4: Dataset S3). The category “sulfur metabolism” also exhibited signatures of amino acid bias (Bray-Curtis distance; Fig. 3), suggesting selection for this functional capacity occurred at the levels of both gene complement and amino acid composition.

**Fig. 4:**
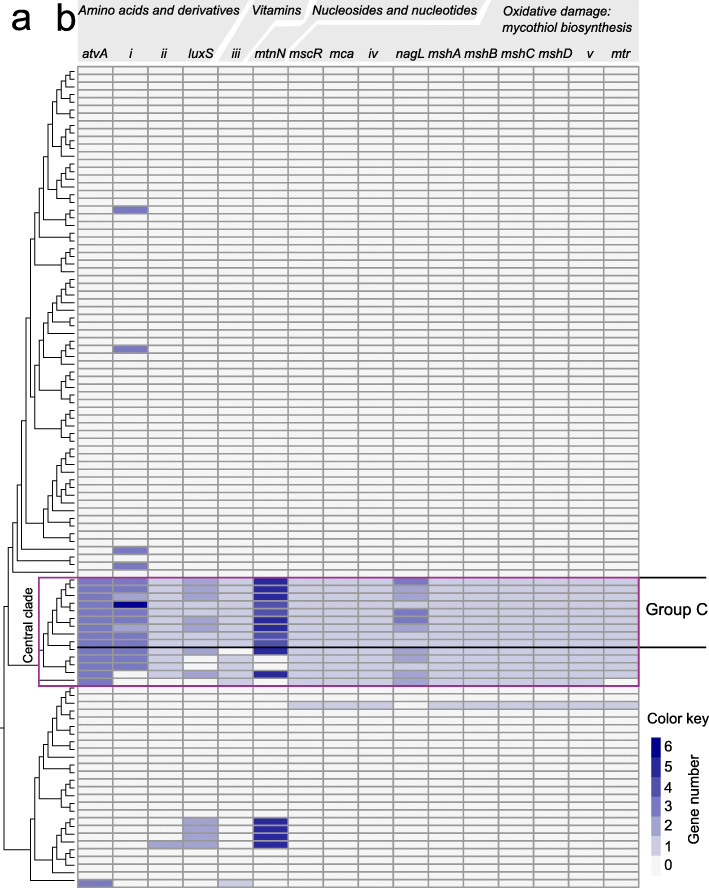
*Arthrobacter* genes typifying the functional potential of Group C. **a** Maximum likelihood *Arthrobacter* phylogenomic tree as for Fig. 1. **b** Heat map of the representation of specific genes in *Arthrobacter* genomes, highlighting those present in Group C and the central clade. *i*, branched-chain acyl-CoA dehydrogenase; *ii*, enoyl-CoA hydratase; *iii*, biotin repressor; *iv*, hydrolase in cluster with formaldehyde/S-nitrosomycothiol reductase; *v*, mycothiol-dependent formaldehyde dehydrogenase

Functional assessments were extended to identify specific genes unique to Group C. A number of genes involved in the synthesis of amino acids, vitamins, and nucleosides were present in all Group C genomes (Fig. 4). The specific genes also tended to be present in the other four, non-Group C members (Group A and Group B) of the central clade (Fig. 4), but had low representation in other *Arthrobacter* genomes (Fig. 4). The most marked feature was a complete mycothiol (MSH) biosynthesis pathway that was present in all Group C genomes (Fig. 4); MSH is a redox-active thiol, functionally analogous to glutathione (which is typically absent from Actinobacteria), that maintains intracellular redox balance and can therefore protect against oxidative damage [42]. Furthermore, MSH potentially serves as a stable reservoir of carbon and sulfur in bacteria [43]. The ability to respond effectively to oxidative damage may be an important trait of microorganisms from cold environments, particularly for facilitating growth at low-temperature limits [44–48]. The MSH pathway was also present in the other four, non-Group C members of the central clade, plus one other Group B member (Fig. 4). Therefore, the MSH pathway plus the individual genes involved in the synthesis of amino acids, vitamins, and nucleosides are characteristic of Group C *Arthrobacter*, but are not unique to this group. If MSH or the other individual genes fulfill roles in facilitating growth at low temperature, the genes may be under stronger positive selection in Group C, while also being retained within Group A and Group B populations (pan genome), but at a significantly lower level.

A total of 48 Group C gene families had significantly higher, and 66 had significantly lower average gene copy number compared to Group A, including four which were absent in Group C genomes (Additional file 5: Dataset S4, *p* < 0.05). The absence of two specific genes is noteworthy: adenosylhomocysteinase, which hydrolyzes S-adenosyl-l-homocysteine (a product of methyl transfer reactions that involve S-adenosyl-l-methionine) to homocysteine and adenosine [49], and formate–tetrahydrofolate (THF) ligase, which catalyzes the initial recruitment of single carbon units for THF-mediated one-carbon metabolism [50]. The absence of both genes would be expected to disrupt the synthesis of methionine from homocysteine, and instead favor the alternative pathway of synthesizing methionine from cysteine; the latter pathway may be connected to MSH metabolism, in that accumulation of cysteine (a precursor of MSH synthesis) is toxic to cells [43], so surplus cysteine not required for MSH synthesis, or resulting from MSH degradation, could be directed to methionine synthesis (Additional file 5: Dataset S4).

Some of the gene families had particularly high copy numbers per genome (~30 in Group A) with large reductions (~6) in Group C (Additional file 5: Dataset S4); this trend was observed for 3-oxoacyl-[acyl-carrier protein] reductase (FabG), glycerate kinase (GlxK), and alcohol dehydrogenase (Adh). For FabG, this likely reflects a reduced capacity of Group C to catalyze the formation of long-chain fatty acids (Additional file 5: Dataset S4). GlxK is an important catabolic enzyme, in that diverse substrates are degraded to glycerate, and GlxK links these degradation pathways to central carbon metabolism [43]. Decreased copy numbers of Adh likely indicates decreased capacities to utilize alcohols. Thus, decreases in GlxK and Adh might reflect decreased substrate preferences by these *Arthrobacter*. It was noteworthy that the copy number of cold shock protein (*csp*) genes was lower in Group C. While *csp* genes are sometimes equated with an ability to grow in the cold or survive cold shock, these nucleic acid binding proteins can perform diverse roles in cellular function (reviewed in Ref. [34]); the findings here reinforce the notion that *csp* and other “stress” genes are not good molecular markers for identifying psychrophiles [5, 33].

### Ecology of Group C *Arthrobacter*

**Fig. 5:**
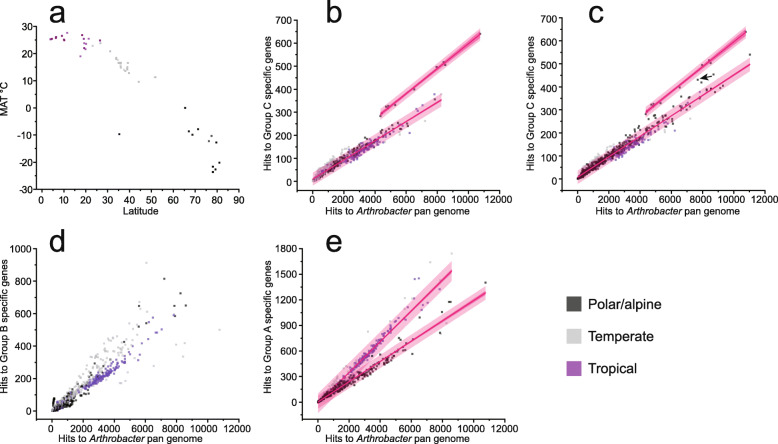
Metagenome analysis of Group C *Arthrobacter*. **a** Depiction of the mean annual temperature (MAT) of surface air at a height of 2 m (European Centre for Medium-Range Weather Forecasts) relative to latitude. The 639 metagenomes are divided into thermal categories: PA (black squares, 196 metagenome), temperate (gray squares, 243 metagenomes), and tropical (purple squares, 200 metagenomes). **b** Linear regression showing the correlation of the abundance of Group C-specific genes within each of the 639 metagenomes (see panel **a**) relative to the abundance of Group C-specific genes within the *Arthrobacter* pan genome. The 95% prediction interval (dark pink band) and 95% confidence interval (light pink band) are shown for each regression line (panels **b**, **c**, and **e**). The upper cluster contains 11 Axel Heiberg Island permafrost metagenomes. **c** As for panel **b**, except with the addition of 334 permafrost metagenomes (total 973 metagenomes). The Stordalen Mire (Abisko, Sweden) metagenome is shown by an arrow. **d** As for panel **b**, except showing Group B-specific genes. **e** As for panel **b**, except showing Group A-specific genes present in PA genomes (lower line) and NPA genomes (upper line). The regression line for the 11 Axel Heiberg Island permafrost metagenomes is not shown

We hypothesized that if the laboratory-generated growth data (Fig. 2) and genomic traits (Fig. 3) translated to competitiveness in cold environments, Group C *Arthrobacter* would be over-represented in metagenome data from PA vs NPA environments. The relative abundance of *Arthrobacter* in environmental samples (publicly available metagenome data) tends to be low, with no metagenome-assembled genomes (MAGs) present in the ~8000 MAGs that were constructed from ~1500 metagenomes [51], and a total of 12 (> 90% completeness) present in 76,831 Integrated Microbial Genomes (IMG) MAGs (December 2019). To facilitate metagenome analyses, group-specific genes (Additional file 6: Dataset S5) were examined in 639 metagenomes representing PA, temperate, and tropical environments (Additional file 7: Dataset S6), with representation shown relative to the *Arthrobacter* pan genome (Fig. 5a) (see the “Methods” section for a description of the analytical approach).

Group C-specific genes were more highly represented in 11 permafrost metagenomes (Fig. 5b). All of the 11 metagenomes came from a single site: Axel Heiberg Island, Nunavut, Canada [52]. The Axel Heiberg Island study reported 76 metagenomes derived from 1-m cores that were used during a controlled thawing experiment [52]. Most Group C-specific genes were enriched in the 65-cm depth active-layer (7 metagenomes), with one from the 35-cm active-layer and three from the 80-cm permafrost-layer (Additional file 1: Table S2). A total of 94% of the variability that exists in hits to Group C for the 639 metagenomes (Fig 5b) was traced to pre-existing variability in hits to the *Arthrobacter* pan genome, and when this covariance was removed by ANCOVA analysis, a statistically significant difference in the y-intercepts for the regression lines (*p* < 0.0001) remained; this confirms the over-representation of Group C-specific genes in the 11 metagenomes compared to the remaining 628 metagenomes.

To assess whether Group C *Arthrobacter* were generally enriched in permafrost regions, all other publicly available permafrost metagenomes (334 metagenomes) were analyzed (Fig. 5c). A number appeared somewhat enriched in Group C-specific genes (e.g., a metagenome from Stordalen Mire near Abisko, Sweden; marked by an arrow in Fig. 5c), but as the slopes of the two regression lines were not parallel, it was not valid to compare the y-intercepts, and hence, the significance of the difference between them could not be evaluated [53].

For Group B-specific genes, no obvious trends separated the PA from NPA metagenomes (Fig. 5d). However, the distribution of Group A-specific genes clustered according to climate classification, with PA metagenomes showing lower Group A content (y = 0.1202x + 3.4193, R^2^ = 0.96801) compared to temperate and tropical metagenomes (y = 0.1804x − 15.015, R^2^ = 0.96989) (Fig. 5e). The 11 Axel Heiberg Island metagenomes had a statistically significant under-representation of Group A-specific genes compared to all other metagenomes (ANCOVA, *p* < 0.0001). This pattern indicates there is selection against Group A *Arthrobacter* in PA environments and/or selection for Group A in NPA environments.

To define variables that may explain the niche adaptation of Group C in the Axel Heiberg Island permafrost, available abiotic and biotic data were used from the permafrost study [13, 14]. A range of physicochemical data were available for each of the four depths (5, 35, 65, and 80 cm), but as the timing of sampling for physicochemical data (0, 4, 6, 8, 11, and 12 weeks) did not align with the timing of sampling for the metagenomes (0, 0.25, 6, 12, and 18 months), the physicochemical data were ultimately not useful for interpreting Group C distribution. Depth, treatment group, and sample core did not explain the variation in species composition across the sites, and although incubation time had some explanatory power for the distribution of the entire permafrost study dataset (data not shown), the metagenomes enriched in Group C *Arthrobacter* were widely distributed and did not cluster together, suggesting the importance of specific microniches in the enrichment of these species. Assessment of the functional potential of the microbial communities in each of the 76 metagenomes using the presence/absence of KO groups also did not identify any significant functional differences (PERMANOVA, *p* > 0.05; data not shown).

In contrast, strong taxonomic associations were identified with many members of the microbial community. Analyses were performed to assess taxa that correlated with Group A and Group C, just Group A, and just Group C (Additional file 8: Dataset S7). A total of 107 operational taxonomic units (OTUs) positively correlated with Group C *Arthrobacter*, and 63 negatively correlated (Additional file 8: Dataset S7). Of the 107 OTUs positively correlated to Group C, 72 were also positively correlated to Group A above the threshold of 0.5 (the remainder were positively correlated with values 0.359–0.499), and no OTUs were positively correlated to Group A that did not also correlate to Group C. The positively correlating OTUs were dominated by both spore- and non-spore-forming members of Actinobacteria and Firmicutes, as well as members of Proteobacteria; the majority of these OTUs were isolated from soil. Negatively correlating bacterial OTUs mainly belonged to marine or lacustrine members of Bacteroidetes, Cyanobacteria, and Proteobacteria, as well as certain eukaryotes (fungi, plants, marine annelid worm).

## Discussion

Numerous studies have been performed to define the critical traits of a psychrophile, including those that have compared genomes that represent a broad range of species and thermal environments (discussed in Ref. [33, 34, 54]). The current study explored genomic characteristics of a lineage with less than 3.5% difference in 16S rRNA gene identity. The analyses revealed that the *Arthrobacter* lineage contains a clade with members (Group C) possessing a clear capacity to grow faster than their relatives (Group A and B) under laboratory growth conditions at −1 °C (Fig. 2). A number of genomic characteristics that potentially explain the physiological capacity of Group C were identified. (1) Group C possess an amino acid composition that is predicted to reduce the stability of a large proportion of proteins thereby enhancing enzyme activity at low temperature [53]. (2) Group C genomes are enriched in sulfur metabolism genes, and sulfur is required for the cysteine component of mycothiol. The synthesis of mycothiol may potentially protect Group C *Arthrobacter* against oxidative damage that may otherwise accumulate as cell division decreases towards the lower temperature limit of growth [44]. (3) Group C exhibits a relatively high proportion of mobile elements, which is a trait shared with some other cold-adapted microorganisms [38–40]. Collectively, the physiological and genomic traits appear compelling for denoting Group C, a cold-adapted clade of *Arthrobacter*.

However, from assessing available metagenome data, we infer that these traits do not translate to a generally enhanced ability to compete in low-temperature environments. Other than the 11 specific Axel Heiberg Island permafrost metagenomes, Group C *Arthrobacter* were not highly represented in the other metagenomes from cold environments, including 144 from Arctic peat soil, 22 associated with glaciers, 42 from polar deserts, and importantly, 365 from other permafrost environments. Even at the Axel Heiberg Island site, Group C-specific genes were not highly abundant at 5- and 20-cm depths. Instead, the pattern of abundance of Group C appears to derive not just from low temperature, but from niche-specific conditions.

Attempting to identify specific niche conditions is not trivial. For the Axel Heiberg Island study, the permafrost microbial community was reported to be dominated by Actinobacteria and Proteobacteria, with significant increases at depth for Firmicutes and Actinobacteria and significant decreases for Acidobacteria, Proteobacteria, and Verrucomicrobia [14]. However, despite these taxonomic differences, we did not identify significant predicted functional differences by depth. When we turned to specifically correlating the abundance of Group C to OTUs from the metagenome data, a large number of OTUs with positive or negative correlations were identified (Additional file 8: Dataset S7). At a broad level, the environmental data of the positively correlating taxa are consistent with Group C associating with other soil bacteria. While this provides scope for investigating specific taxa that may help shape the niche that Group C occupy, determining which taxa are important and the nature of their interactions will require a dedicated effort.

For the negatively correlating cohort, they tend to represent isolates from non-soil environments (Additional file 8: Dataset S7) and may therefore represent non-indigenous microorganisms that have been introduced. The permafrost samples were obtained from an “upland polygonal terrain in proximity to the McGill Arctic Research Station at Expedition Fjord (79°24’57"N, 90°45’46"W)” [13]. The prevalence of negatively correlating OTUs matching to Proteobacteria isolated from sea water may reflect aeolian carriage from Expedition Fjord, which is located ~8 km from the Research Station. As the samples were obtained for a simulated permafrost-thaw experiment [52], the negatively correlating OTUs may also reflect environmental disturbance.

## Conclusions

Our study commenced with the analysis of genome sequences of new Group C *Arthrobacter* isolated from the Tibetan Plateau and progressed through to a rationalization of Group C abundance in global metagenomes. Group C was clearly distinguished from Group A *Arthrobacter* by possessing genomic signatures consistent with its representation in PA environments and an ability to grow faster when cultivated at −1°C. Assessment of available metagenome data points to the Group C traits as being more relevant to cold niches rather than competitiveness across global permafrost or cold soil environments. The challenge in being able to define the specific niche parameters enabling Group C *Arthrobacter* to be relatively competitive illustrates the inherent difficulties associated with defining “cause and effect” for explaining “why” microorganisms reside in the environments in which they are found, that is, the characteristics of the ecological niches that define microbiome structure [1]. Without knowing the specific effectors, the ability to understand and predict responses to environmental changes is greatly compromised [5, 7, 55, 56]. Establishing long-term data records that include comprehensive metadata associated with monitoring sites, including metadata for each biological sample, will be essential for learning how to link environmental parameters to microbial processes. In a study of sulfate reduction in Arctic marine sediments, growth yield was reasoned to be the most relevant factor for determining the competitiveness of sulfate-reducing bacteria in permanently cold marine sediments [54]. These findings illustrate that for cold environments, linking genomic and metagenomic data to measurements of metabolic rates, growth rates, and growth yields will undoubtedly help to clarify how specific microbial processes and associated taxa are influenced by environmental temperature.

While the characteristics that define the Group C niche are still to be defined, at sites where Group C *Arthrobacter* are relatively abundant, they may have value as a biomarker for monitoring the stability of those locations. Moreover, Group A *Arthrobacter* may serve as a more broadly useful biomarker of soil microbial communities. Group A exhibited high relative abundance across NPA metagenomes and relatively low abundance across PA metagenomes. As the data indicate environmental temperature exerts a broad, strong influence on Group A *Arthrobacter*, we predict that environmental warming will generally increase the relative abundance of Group A. Similar influences of environmental temperature have been described for the marine SAR11 clade, including the predicted displacement of polar specialists by phylotypes from warmer latitudes [57]. Depending on how strongly the environmental factors other than temperature select for Group C in permafrost, the apparent broad influence of temperature on Group A suggests it will displace Group C from the niches in which it is currently relatively competitive.

## Methods

### *Arthrobacter* isolation and genome sequencing

Sampling and isolation of *Arthrobacter* from lakes, glaciers, and a wetland on the Tibetan Plateau was performed based on procedures previously described [26, 27, 58, 59], and information associated with sampling and isolation is provided in Additional file 1: Table S1. Briefly, surface water samples from lakes Dawa Tso, Gomang Tso, Peng Tso, Ranwu, Sumzhi Tso, Yamdrok Tso, and Zigetangcuo were collected during the 2012 summer fieldwork based on procedures previously described [58]. All water samples were collected in sterile 250-mL Nalgene bottles and stored in the field at 4 °C. After transport at 4 °C to the Institute of Tibetan Plateau Research-Lhasa, 15% glycerol (v/v) was added and the samples were stored at −20 °C prior to transport and storage of samples at −20 °C at the Institute of Tibetan Plateau Research-Beijing. For glacier samples, 12-cm-diameter ice cores were drilled in Noijinkangsang (August 2007; 33-m-long ice core) and Ulugh Muztagh (May 2012; 164-m-long ice core) glaciers. The ice cores were cut into sub-sections with intervals of 5–10 cm using a bandsaw in a walk-in-freezer (−20 °C). Ice on the surface of the samples (1 cm thick) was chipped off using a sterilized blade, the inner cores were rinsed with cold ethanol (95%), followed by cold, triple-autoclaved, double-distilled water. The frozen lake water and the ice core samples were placed in autoclaved containers and melted slowly at 4 °C before being used for cultivation attempts. A volume of 200 μL of thawed water of each sample was placed directly onto R2A solid medium for cultivation.

Soil cores at 0–5-cm soil depth were collected from the Madoi wetland in August 2011 [59]. Soil samples were placed in a box with ice packs during transportation and were stored in the laboratory at 4 °C. The soil samples were suspended in triple-autoclaved, double-distilled water (m/v, 1:10), incubated statically at room temperature for 2 h, and 200 μL of supernatant was dispensed directly onto R2A solid medium for cultivation.

All cultivation on R2A solid medium (lake, glacier, and wetland samples) was performed at the Institute of Tibetan Plateau Research-Beijing in incubators at temperatures ranging from 4 to 24 °C for a period ranging from 1 week to 2 months (Additional file 1: Table S1). Colonies were quadrant-streaked several times for purification, and purity was assessed using microscopy.

Genomic DNA was extracted from isolates using a TIANamp Bacteria DNA Kit (Tiangen, Beijing) following the manufacturer’s instructions. The 16S rRNA genes were amplified with the universal bacterial primers 27F (5′AGAGTTTGATCCTGGCTCAG-3′) and 1492R (5′CGGTTACCTTGTTACGACTT-3′) and the amplification products were sequenced at Boai Yonghua (Beijing) on an ABI PRISM 3730xl sequencer. The taxonomy of the isolates was determined by aligning the 16S rRNA gene sequences against the NCBI-nr nucleotide database using blastn (Blast+ v2.9.0). The 16 *Arthrobacter* isolates (Additional file 1: Table S1) were deposited in the China General Microbiological Culture Collection Center (CGMCC) with accession numbers: CGMCC 1.16187-1.16198, 1.16223, and 1.16312.

Using genomic DNA (extracted as described above) for the 16 isolates, paired-end libraries with an insert size of 500 bp were constructed and sequenced using an Illumina Hiseq 2000 platform. Prior to de novo sequence assembly, low-quality reads were filtered out using Fastp with default options [60]. Filtered sequencing reads were subjected to assembly using SPAdes v3.11.1 with default options [61]. The assembled genome sequences were deposited in DDBJ/ENA/GenBank under the BioProject PRJNA421662.

For genomic analyses, three of the 16 isolates were excluded due to genome quality or dereplication criteria (also see the “Preparation of Arthrobacter genomes for analysis” section below and the “Phylogenomics” section in the “Results” section). All 13 isolates used for the study represented unique sites (lakes, glaciers, or wetland) or specific location of an individual lake (Zigetangcuo) or depth of a glacier core (Ulugh Muztagh) (Additional file 1: Table S1).

### Growth temperature response

Three replicates of each *Arthrobacter* were grown in 100 mL of R2A broth in 150-mL flasks at 25, 5, and −1°C for up to 10 days. The optical density was measured at 600 nm (OD_600_) using a Microplate Reader (MD, SpectraMax M5) by transferring 200 μL of the culture into microwells. OD_600_ measurements were taken every 24 h for cultivation at −1 °C and 5 °C, and every 12 h for cultivation at 25 °C. For cultivation at −1°C, flasks were placed in ice produced by an ice maker (TKKY, FM40) with flasks placed in a ~ 4°C refrigerator and ice replaced every 12 h. Growth at 5 and 25°C was performed using a constant-temperature incubator as described previously [62]. All cultures were grown statically, with flasks swirled to resuspend biomass prior to recording OD_600_. *Arthrobacter* used for growth temperature profiles were as follows: Group A: *A. luteolus*, *A. globiformis*, and *A. subterraneus*; Group B: *Arthrobacter* sp. 4R501, *Arthrobacter* sp. 9E14, and *Arthrobacter* sp. 08Y14; and Group C: *A. alpinus*, *Arthrobacter* sp*.* A3, and *Arthrobacter* sp. N199823.

### Preparation of *Arthrobacter* genomes for analysis

As the taxonomic assignment of the genus *Arthrobacter* is not consistent, in August 2018, all genome sequences with the taxonomy identifier “Arthrobacter” or “Micrococcaceae” were retrieved from GenBank, providing a total of 427 genomes including the 16 new Tibetan Plateau genomes. The completeness of each genome was calculated using CheckM v1.0.7 with default options [63]. Genomes composed of > 300 contigs, with an N50 of < 20 kb, completeness of < 95%, and contamination > 5% were removed. Genomes were dereplicated to remove genomes with an AAI ≥ 99.5%. AAI values were calculated using CompareM with default options (https://github.com/dparks1134/CompareM). ANI was calculated using the ANI calculator (http://enve-omics.ce.gatech.edu/ani/). A total of 210 genomes met quality requirements, which included 13 of the 16 new Tibetan Plateau genomes (Additional file 2: Dataset S1). Gene families were clustered using FastOrtho software (--pv_cutoff 1-e5 --pi_cutoff 70 --pmatch_cutoff 70) (http://enews.patricbrc.org/fastortho/) with the cutoff values set according to Parks et al. [35]. A gene family matrix was produced using custom PERL scripts, and non-functional-based group-specific genes were calculated based on this matrix. The annotation of genes was standardized by annotating all genomes using RAST (Rapid Annotation using Subsystem Technology) [64] and PROKKA [65].

### Phylogenetic and genomic analyses

For phylogenomic clustering, *Cellulomonas carbonis* T26 and *C. fimi* ATCC 484 were chosen as the outgroup as they are close relatives of Micrococcaceae [66], and species that are closely related to the in-group are more suitable for phylogenetic reconstruction than distantly related species [67]. A maximum likelihood phylogenomic tree was constructed using PhyloPhlAn2 with default options [68]. A Bayesian tree was constructed using MPI Mrbayes v3.2 (prset aamodelpr = mixed, mcmc nchains = 16, ngen = 300,000, and leaving other parameter values as default) [69]. The F measure (harmonic mean of precision) provides a metric for determining if taxa are operationally monophyletic (F measure ≥ 0.95) [35] and was calculated as F = 2 × ((precision × recall)/(precision + recall)). The genome-wide amino acid composition was calculated using CompareM with the function aa_usage. The stability curves of proteins were predicted by SCooP [37] using the PDB (Protein Data Bank) files modeled by SWISS-MODEL [70]. The stability equations of the same protein from different hosts were visualized and smoothed using ggplot2 v3.2.1 [71]. The stability curves were analyses for 180 single-copy genes that were shared by most genomes; a small number of genomes had multiple copies of genes, and up to three genomes were permitted to have the absence of the gene in order to account for the use of unclosed genomes (99 of the 106). After retrieval from SWISS-MODEL of all possible PDB files matching the candidate genes, a total of 17,339 stability equations were generated (Additional file 3: Dataset S2). Ordination and statistical analyses, including three-dimensional nonmetric multidimensional scaling and gene enrichment analyses, were performed with R v3.3.3 and Origin v9.5. For comparisons between Group A, B, and C *Arthrobacter*, group-specific genes or functions were defined as being present in 95% of the target group (e.g., Group C) genomes and absent in 95% of each of the other group(s) (e.g., Group A) genomes. Group-specific genes were identified (Group A, 74 genomes, 16,149 specific genes; Group B, 22 genomes, 4675 specific genes; Group C, 10 genomes, 969 specific genes; Additional file 6: Dataset S5) and normalized to account for the different number of genomes used for each group. To account for differences in gene content between *Arthrobacter*, comparisons were calculated relative to the total *Arthrobacter* gene complement from all 106 *Arthrobacter* genomes (referred to as the *Arthrobacter* pan genome). Gene copy number was calculated as the average number of the gene for each genome in a group (e.g., Group C), with gene loss or gain calculated from the average copy number for the groups (e.g., Group C vs Group A). To assess the bias of amino acid composition of different functional classes of proteins, genes were assigned to functional categories (assigned by RAST) and total amino acid composition for all proteins from the functional category was compared between groups (e.g., Group C vs Group A). Similarity was measured by Bray-Curtis distance with larger Bray-Curtis distances denoting stronger bias, possibly indicative of selection pressure [41]. The functional potential of groups was also compared using enrichment analysis [41]. Briefly, the presence or absence of KEGG Ortholog (KO) groups in genomes and metagenomes (see the “Collection and analysis of metagenomes” section) was assessed [41], and non-parametric one-way ANOVA was used to identify differentially abundant categories using R [72].

### Collection and analysis of metagenomes

Assembled metagenomes were downloaded from IMG (https://genome.jgi.doe.gov/portal/). Classification into PA or NPA environments were made using metadata associated with metagenomes, supplemented by Köppen-Geiger climate classifications (to define temperate and tropical regions) using ArcGIS location data [73]. Analyses were initially performed using 639 metagenomes from environments with MAT ranging from −24 to 28 °C, representing PA (*n* = 196, gene count = 183 million), temperate (*n* = 243, gene count = 190 million), and tropical (*n* = 200, gene count = 841 million) zones (Additional file 7: Dataset S6). Subsequently, all additional (334) available (May 2020) unique assembled permafrost metagenomes were analyzed. Analyses assessed the relative abundance of each *Arthrobacter* group (A, B, and C) using group-specific genes (see the “Phylogenetic and genomic analyses” section) by performing a local alignment search against the metagenomes using DIAMOND v0.9.24 with the arguments --outfmt 6, --query-cover 70, --id 70, --evalue 1e-5, and leaving others as default [74]. One-way ANCOVA was used to assess statistical differences between regression lines for groups of metagenomes [75] using the data import webform for k = 2 at http://vassarstats.net/vsancova.html. Correlation analyses were performed between *Arthrobacter* groups and other members of the microbial community from 76 metagenomes derived from a simulated permafrost-thaw experiment [14]. OTUs were assigned from IMG phylodist matches (which are based on the top taxon in the IMG isolate database) by clustering the IMG phylodist matches at the genus level; < 4% of OTUs had < 35% identity. The raw abundance of all OTUs was determined, with 956 meeting the criteria of average abundance ≥ 2, and detection in at least 56 of the 76 metagenomes. The 956 OTUs were used to construct a correlation matrix using SparCC [76] implemented in python3 with default parameters (20 iterations). One hundred simulated datasets were created by random shuffling of the original input with replacement, and their correlation matrices were constructed in the same way. The simulated datasets were used to calculate the one- and two-sided pseudo *p*-values. The selected threshold for strong correlations was > | 0.5 |. To assess if depth, incubation time, treatment group, sample core, or physicochemical data explained the variation in species composition across permafrost sites, a generalized linear latent variable model was employed as implemented in the R package gllvm [77]. All custom scripts are available at https://github.com/environmental-genomes/Arthro.

## Supplementary Information

**Additional file 1: **Tibetan Plateau sampling sites, *Arthrobacter* phylogenetic, genomic, functional and metagenomic analyses. Supplementary figures. **Figure S1.** Tibetan Plateau sampling locations. **Figure S2.** Micrococcaceae phylogenomic trees. **Figure S3.** Comparison of *Arthrobacter* genomic characteristics. **Figure S4.** Comparison of *Arthrobacter* genome-wide amino acid composition. **Figure S5.** Correlation between the content of an amino acid and genome G+C content. **Figure S6.** Protein stability predictions. **Figure S7.** Enrichment analysis of *Arthrobacter* functional potential. Supplementary tables. **Table S1.** Sampling and isolation information associated with Tibetan Plateau *Arthrobacter* isolates. **Table S2.** Depth distribution of the 11 Axel Heiberg Island metagenomes enriched in Group C *Arthrobacter.*

**Additional file 2: Dataset S1.** Micrococcaceae and *Arthrobacter* genomes used in this study.

**Additional file 3: Dataset S2.** Proteins and stability equations used for stability predictions.

**Additional file 4: Dataset S3.** Data used for enrichment analyses of functional categories.

**Additional file 5: Dataset S4.** Gene copy number data.

**Additional file 6: Dataset S5.** Protein sequences of *Arthrobacter* group-specific genes.

**Additional file 7: Dataset S6.** Metagenomes used in this study.

**Additional file 8: Dataset S7.** Correlation of the abundance of *Arthrobacter* with taxa from Axel Heiberg Island metagenomes.

## Data Availability

The 16 newly isolated *Arthrobacter* were deposited in the China General Microbiological Culture Collection Center (CGMCC) with accession numbers: CGMCC 1.16187-1.16198, 1.16223, and 1.16312. The assembled genome sequences for newly isolated *Arthrobacter* were deposited in DDBJ/ENA/GenBank under the BioProject PRJNA421662. All custom scripts are available at https://github.com/environmental-genomes/Arthro.
