# Supplementary material for: Linking genomic and physiological characteristics of psychrophilic Arthrobacter to metagenomic data to explain global environmental distribution
Source: Microbiome. 2021 Jun 12;9:136. doi: 10.1186/s40168-021-01084-z (PMC8196931; doi:10.1186/s40168-021-01084-z)
Supplement: Supplementary file 2 — Additional file 1: Tibetan Plateau sampling sites, Arthrobacter phylogenetic, genomic, functional and metagenomic analyses. Supplementary figures. Figure S1. Tibetan Plateau sampling locations. Figure S2. Micrococcaceae phylogenomic trees. Figure S3. Comparison of Arthrobacter genomic characteristics. Figure S4. Comparison of Arthrobacter genome-wide amino acid composition. Figure S5. Correlation between the content of an amino acid and genome G+C content. Figure S6. Protein stability predictions. Figure S7. Enrichment analysis of Arthrobacter functional potential. Supplementary tables. Table S1. Sampling and isolation information associated with Tibetan Plateau Arthrobacter isolates. Table S2. Depth distribution of the 11 Axel Heiberg Island metagenomes enriched in Group C Arthrobacter. [file 40168_2021_1084_MOESM1_ESM.pdf]

Linking genomic and physiological characteristics of psychrophilic *Arthrobacter* to metagenomic data to explain global environmental distribution

Liang Shen, Yongqin Liu, Michelle A. Allen, Baiqing Xu, Ninglian Wang, Timothy J. Williams, Feng Wang, Yuguang Zhou, Qing Liu, Ricardo Cavicchioli

Additional file 1: Tibetan Plateau sampling sites, *Arthrobacter* phylogenetic, genomic, functional and metagenomic analyses

Supplementary figures

Fig. S1 Tibetan Plateau sampling locations

Fig. S2 Micrococcaceae phylogenomic trees

Fig. S3 Comparison of *Arthrobacter* genomic characteristics

Fig. S4 Comparison of *Arthrobacter* genome-wide amino acid composition

Fig. S5 Correlation between the content of an amino acid and genome G+C content

Fig. S6 Protein stability predictions

Fig. S7 Enrichment analysis of *Arthrobacter* functional potential

Supplementary tables

Table S1 Sampling and isolation information associated with Tibetan Plateau *Arthrobacter* isolates.

Table S2 Depth distribution of the 11 Axel Heiberg Island metagenomes enriched in Group C *Arthrobacter*

Datasets (accompanying excel files)

Dataset S1 Micrococcaceae and *Arthrobacter* genomes used in this study

Dataset S2 Proteins and stability equations used for stability predictions

Dataset S3 Data used for enrichment analyses of functional categories

Dataset S4 Gene copy number data

Dataset S5 Protein sequences of *Arthrobacter* group-specific genes

Dataset S6 Metagenomes used in this study

Dataset S7 Correlation of the abundance of *Arthrobacter* with taxa from Axel Heiberg Island metagenomes

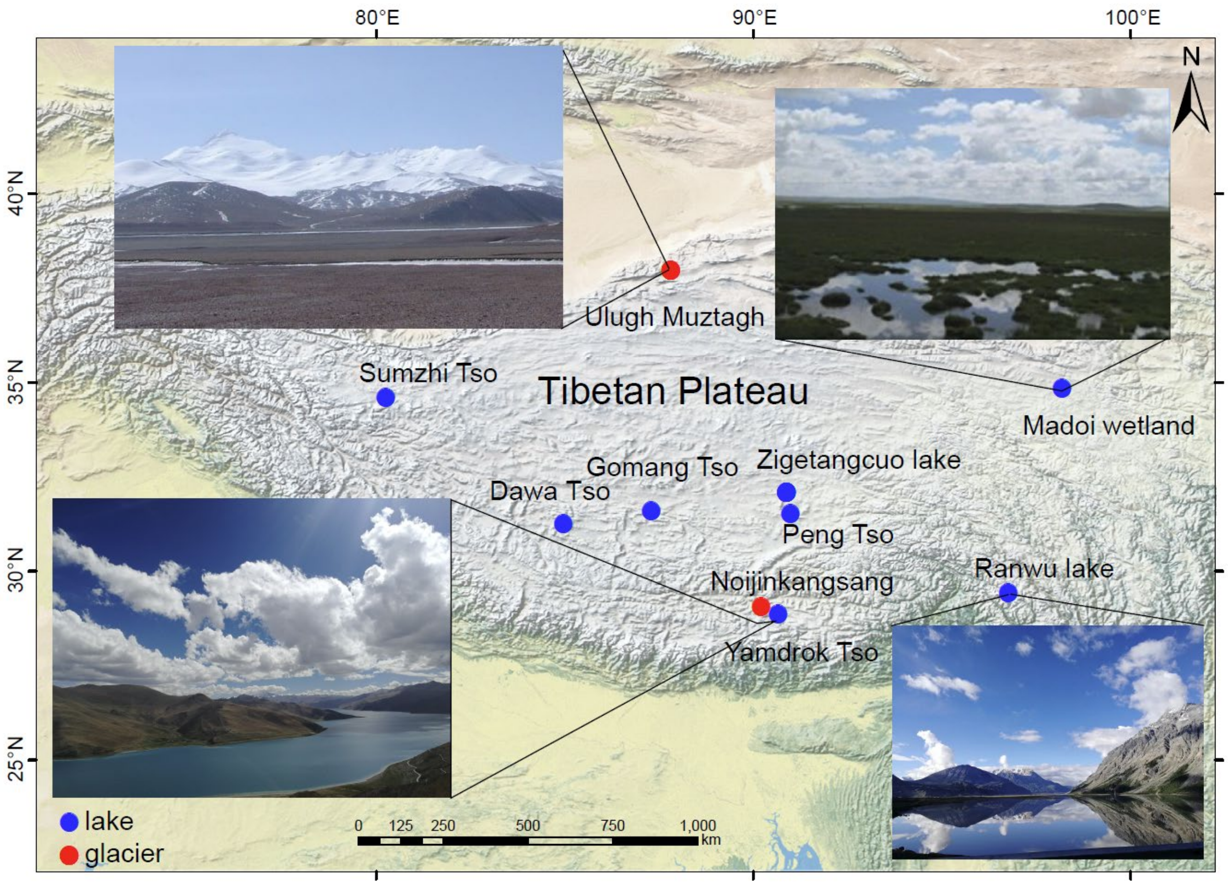

Fig. S1 Tibetan Plateau sampling locations. ArcGIS map showing the Tibetan Plateau with the locations of lake, glacier and wetland sampling sites. Credit to Zhihao Zhang for constructing the ArcGIS map. Photo credits: Wenqiang Wang for Yamdrok Tso and Ranwu lake; Yongcui Deng for Madoi wetland; Ninglian Wang for Ulugh Muztagh glacier.

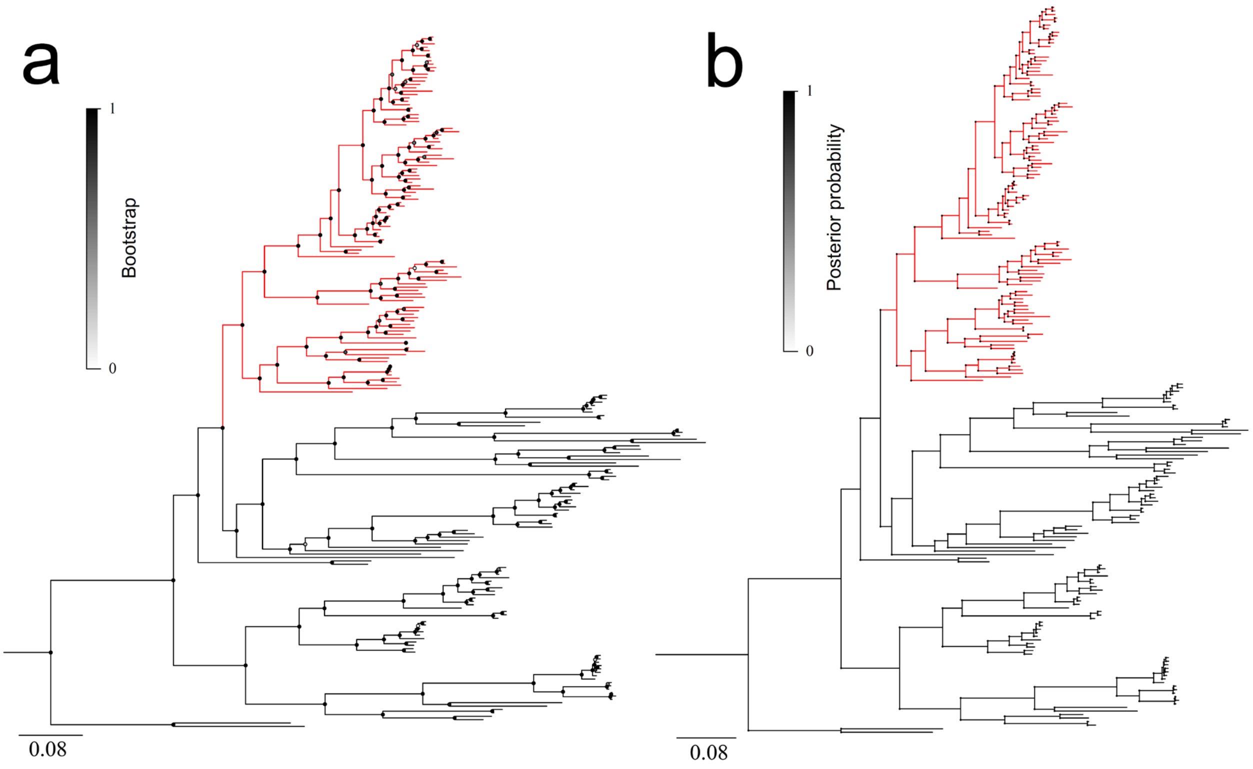

Fig. S2 Micrococcaceae phylogenomic trees. **(a)** ML phylogenomic tree of 210 non-redundant high-quality Micrococcaceae genomes using PhyloPhlAn. **(b)** Bayesian phylogenomic tree constructed by Mrbayes based on concatenated alignment of orthologous proteins from PhyloPhlAn. The upper clade of the ML and Bayesian trees represents *Arthrobacter* strains (red lines). Dots at nodes indicate bootstrap percentages for the ML tree, and posterior probabilities for the Bayesian tree. Bar, 0.08: accumulated changes per amino acid for ML and Bayesian trees.

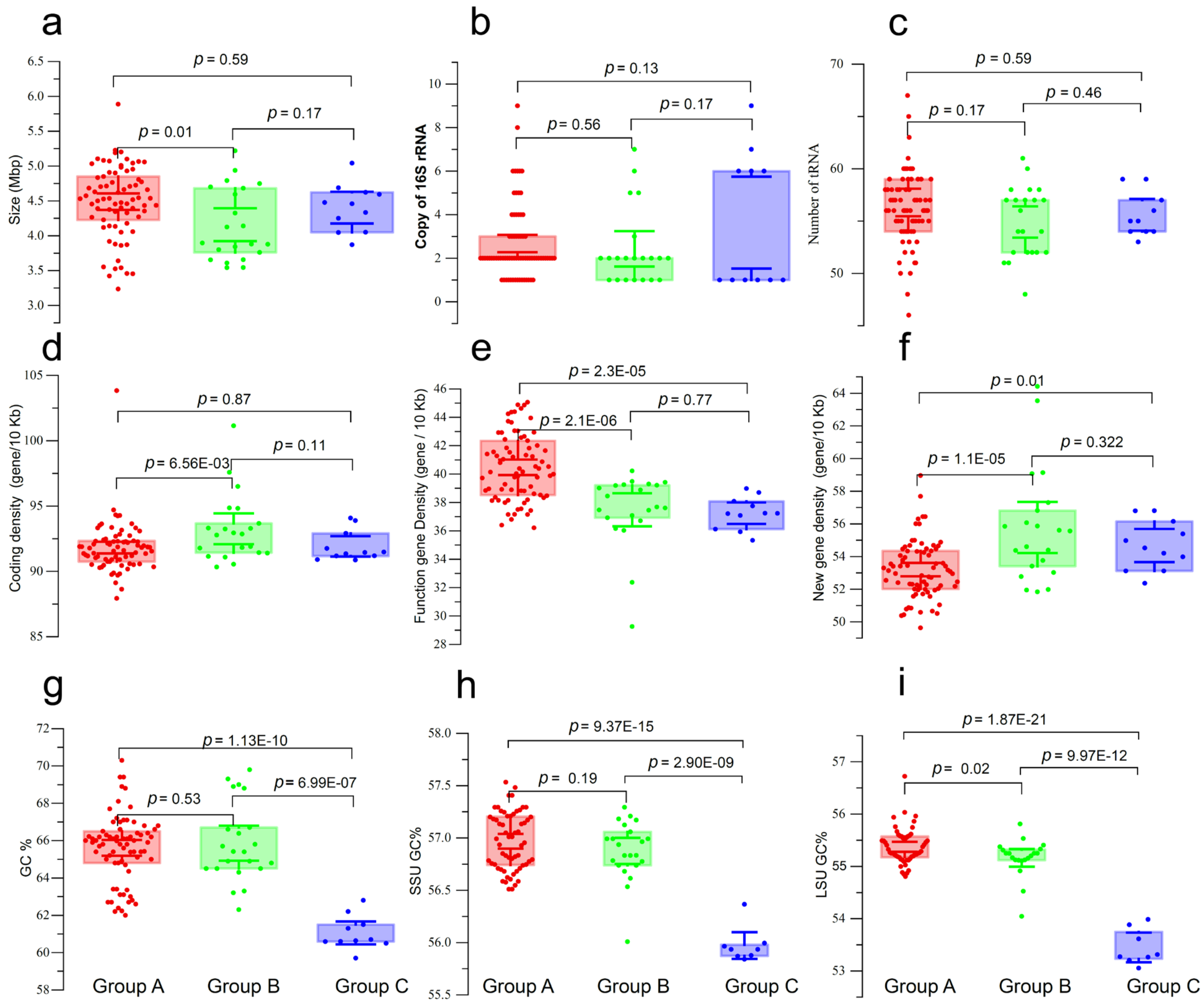

Fig. S3 Comparison of *Arthrobacter* genomic characteristics. Box plots of genomic composition comparing Group A, B and C *Arthrobacter*. **(a)** Genome size. **(b)** 16S rRNA gene copy number. **(c)** tRNA gene copy number. **(d)** Gene coding density calculated as the sum of the length of open reading frames divided by the size of the genome. **(e)** Density of genes with annotated functions, calculated as for (d). **(f)** Density of genes with no annotated functions, calculated as for (d). **(g)** Genome G+C content. **(h)** G+C content of SSU rRNA genes. **(i)** G+C content of LSU rRNA genes. In box plots, boxes represent the interquartile range with horizontal lines showing maximum and minimum values, excluding outliers.

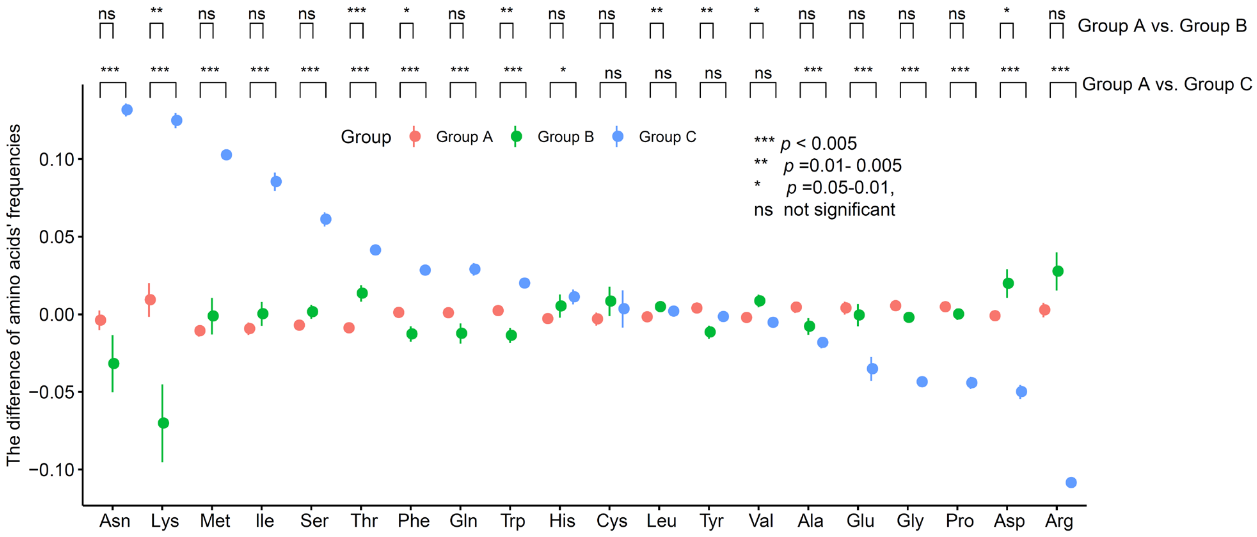

Fig. S4 Comparison of *Arthrobacter* genome-wide amino acid composition. The mean difference (colored circles) and standard deviation (vertical bars drawn through circles) are plotted. *P* values were obtained using One-way ANOVA, with significance indicated above the plot for Group A vs Group B, and Group A vs Group C.

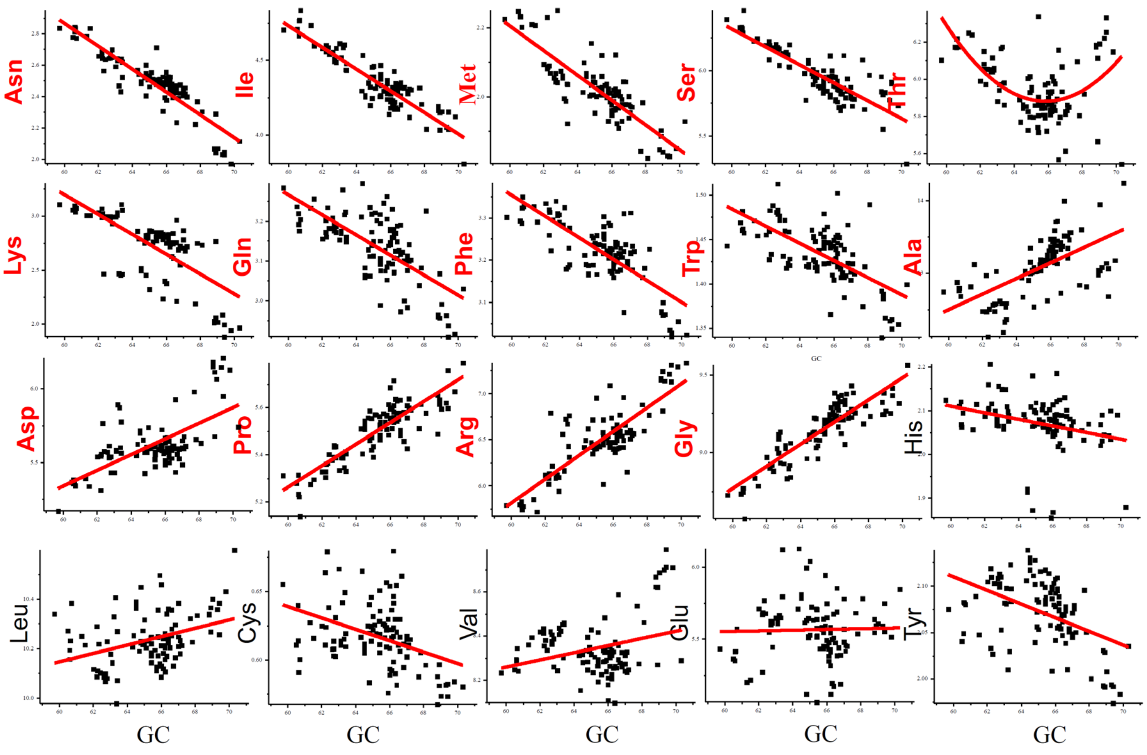

	Asn	Ile	Met	Ser	Phe	Lys	Gln	Gly	Pro	Arg
Number of Points	106	106	106	106	106	106	106	106	106	106
Degrees of Freedom	104	104	104	104	104	104	104	104	104	104
F Value	458.00	444.94	257.26	245.78	154.01	102.81	87.71	344.20	297.36	217.95
R-Square (COD)	0.81	0.81	0.71	0.70	0.60	0.50	0.46	0.77	0.74	0.68
Adj. R-Square	0.81	0.81	0.71	0.70	0.59	0.49	0.45	0.77	0.74	0.67
P Value	0	0	0	0	0	0	1.776E-15	0	0	0

	Asp	Trp	Ala	Cys	Glu	His	Leu	Val	Tyr	Thr
Number of Points	106	106	106	106	106	106	106	106	106	106
Degrees of Freedom	104	104	104	104	104	104	104	104	104	103
F Value	74.51	76.98	66.83	20.13	0.08	9.32	15.68	9.35	27.80	25.3203
R-Square (COD)	0.42	0.43	0.39	0.16	0.00	0.08	0.13	0.08	0.21	0.33
Adj. R-Square	0.41	0.42	0.39	0.15	0.00	0.07	0.12	0.07	0.20	0.32
P Value	7.516E-14	3.642E-14	7.634E-13	1.876E-05	0.77379	0.00288	0.0001375	0.00284	7.364E-07	1.14E-09

Fig. S5 Correlation between the content of an amino acid and genome G+C content. Linear regression showing the correlation of each of the 20 amino acids vs genome G+C content for each of the 106 *Arthrobacter* genomes. Amino acids are specified on the right hand y-axis of the plots, with $R^2 < 0.3$ (black font) and $R^2 > 0.3$ (red font). Statistical analyses were performed using Origin 2018 with the outcomes of the linear regression shown below the plots.

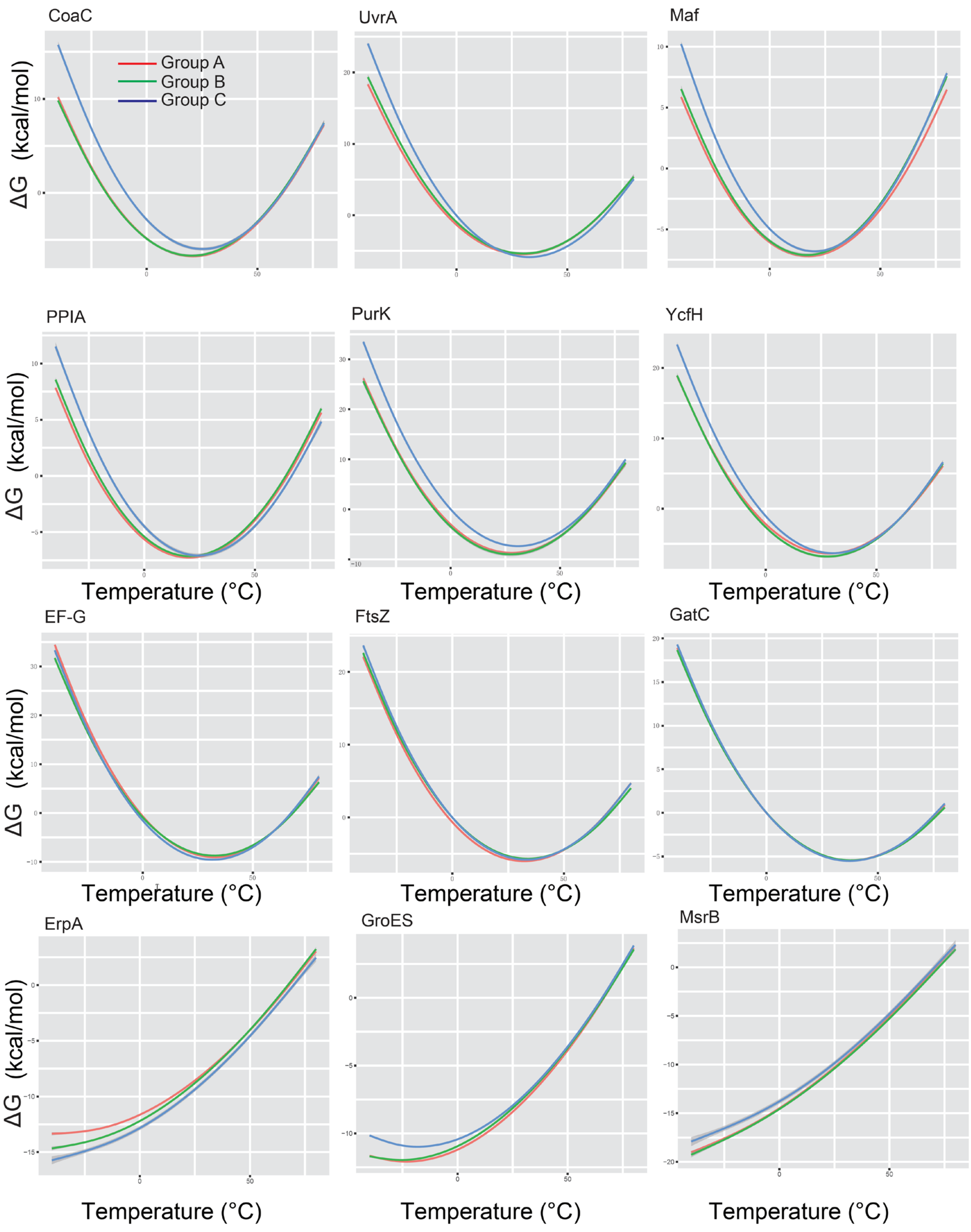

Fig. S6 Protein stability predictions. Predicted stability curves were generated using the SCooP algorithm. Robust stability curves were generated for 86 of the 180 proteins examined. Shown are examples of Group C proteins with higher ΔG (less stability) at -1 °C (CoaC, UvrA, Maf, PPIA, PurK, and YcfH), very similar stability curves (EF-G, FtsZ, and GatC), and proteins that did not produce meaningful stability curves (ErpA, GroES, and MsrB).

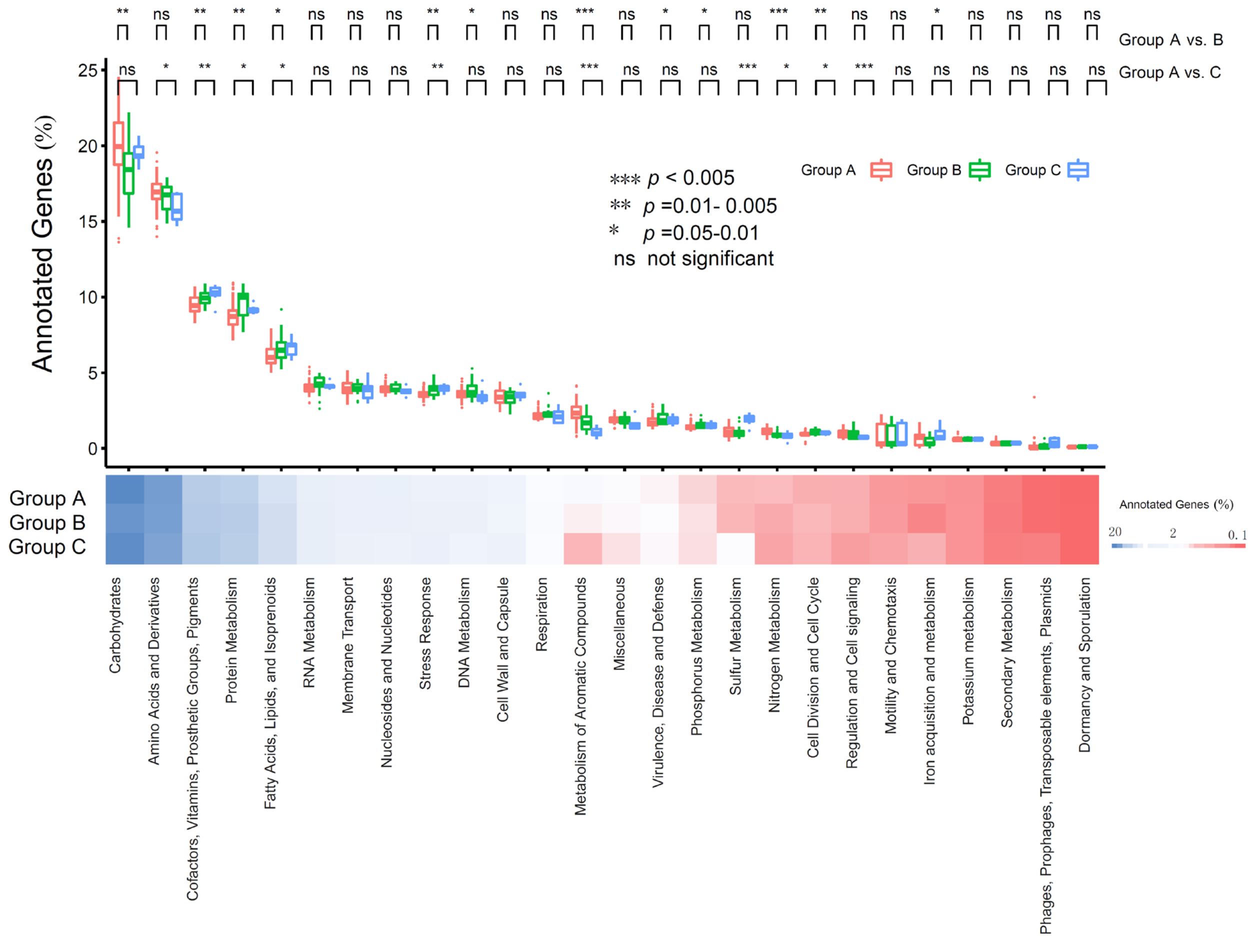

Fig. S7 Enrichment analysis of *Arthrobacter* functional potential. Enrichment analysis was performed on functional gene categories comparing Group A, B and C *Arthrobacter* genomes. The box plots correspond to the abundance of each functional category, ranked in descending order of relative abundance from right to left along the x-axis. Significance (*P* values) were obtained using one-way ANOVA. The heat map depicts the average percentage of number of genes for each functional category for each group.

Additional file 1: Table S1 Sampling and isolation information associated with Tibetan Plateau *Arthrobacter* isolates.

***Arthrobacter* sp. isolated[1]**	**Location[2]**	**Latitude, Longitude[3]**	**Sample[4]**	**Date[5]**	**Temp. ºC[6]**	**Lake water[7]**					**Isol. temp. ºC[8]**
						**Salinity g/L**	**pH**	**Cond. µS/cm**	**Chl-a µg/L**	**DO mg/L**	
08Y14	Yamdrok Tso	28.8521, 90.6506	lake water, 08Y	2012 August	11	1.5	9.3	2400	0.99	9.3	24
4R501	Ranwu lake	29.4167, 96.7500	lake water, 4R5	2012 August	6	0.32	8.1	142	0.27	7.0	10
9E06[9]	Peng Tso	31.5006, 90.9724	lake water, 9E	2012 August	12	8.5	9.9	14700	0.18	6.3	24
9E14[9]	Peng Tso	31.5006, 90.9724	lake water, 9E	2012 August	12	8.5	9.9	14700	0.18	6.3	24
9416[9]	Peng Tso	31.5006, 90.9724	lake water, 9E	2012 August	12	8.5	9.9	14700	0.18	6.3	24
9E18	Peng Tso	31.5006, 90.9724	lake water, 9E	2012 August	12	8.5	9.9	14700	0.18	6.3	24
DWC3	Dawa Tso	31.2434, 84.9646	lake water, DWC3	2012 August	16	19	9.3	30100	2.1	5.7	24
GMC3	Gomang Tso	31.5854, 87.2862	lake water, GMC3	2012 August	14	6.5	9.8	11400	0.8	NA	24
SX1312	Sumzhi Tso	34.6000, 80.2500	lake water, SX13-1	2012 August	12	0.26	8.5	537	1.6	5.8	24
ZGTC131	Zigetangcuo	32.0836, 90.8709	lake water, ZGTC1-3	2012 August	15	14	10	22400	0.83	5.9	24
ZGTC212	Zigetangcuo	32.0836, 90.8709	lake water, ZGTC2-1	2012 August	15	14	10	22400	0.83	5.9	24
ZGTC412	Zigetangcuo	32.0836, 90.8709	lake water, ZGTC4-1	2012 August	15	14	10	22400	0.83	5.9	24
Y81	Madoi wetland	34.8333, 98.1667	wetland soil, Y8	2011 August	13						15
B0490	Ulugh Muztagh glacier	36.4125, 87.3850	ice core, 25.98 m	2012 May	-4						4
B1805	Ulugh Muztagh glacier	36.4125, 87.3850	ice core, 97.07 m	2012 May	-4						4
N199823	Noijinkangsang glacier	29.0400, 90.2000	ice core, 4.71 m	2007 August	-7						4

[1] Designation of *Arthrobacter* isolate.
[2] Location of lake, wetland or glacier (also see Additional file 1: Fig. S1).
[3] Geographic coordinates of the lake, wetland or glacier.
[4] Sample type denoting location on the lake or in the wetland where the sample was taken (e.g., 08Y for isolate 08Y14), or the specific depth of the glacier ice core that was used for *Arthrobacter* isolation (e.g., 25.98 m for B0490).
[5] Date the lake water, wetland soil or glacier ice core was obtained.
[6] Temperature of the sampled lake water or wetland soil, or annual mean air temperature of the glacier from which the ice core was obtained.
[7] Lake water measurements recorded at the time of sampling with a YSI multi-probe Water Quality Sonde (YSI EXO2, Yellow Springs, OH). Temp. (temperature ºC); Cond. (conductivity); Chl-a (chlorophyll-a); DO (dissolved oxygen); NA (not available).
[8] Incubation temperature used for the isolation of the *Arthrobacter* isolates.
[9] The three isolates 9E06, 9E14 and 9E16 (highlighted in gray shading) were not used in the study due to genome quality or dereplication criteria: 9E14 did not pass the quality check as it was composed of > 300 contigs, and because 9E06, 9E16 and 9E18 had an AAI ≥ 99.5% with each other, one of the three isolates was randomly selected for use (9E18).

Table S2 Depth distribution of the 11 Axel Heiberg Island metagenomes enriched in Group C *Arthrobacter*

**Depth (cm)**	**Depth type**	**Number of Group C enriched metagenomes**	**Number of metagenomes for the depth**
5	Active layer	0	19
35	Active layer	1	12
65	Active layer	7	22
80	Permafrost	3	23
